# Callers' perceptions of their contact with a rheumatology telephone helpline

**DOI:** 10.1002/msc.1374

**Published:** 2018-11-23

**Authors:** Susann Arvidsson, Maria L. Nylander, Stefan Bergman

**Affiliations:** ^1^ School of Health and Welfare Halmstad University Halmstad Sweden; ^2^ Spenshult Research and Development Centre Halmstad Sweden; ^3^ Swedish Rheumatism Association Stockholm Sweden; ^4^ Primary Health Care Unit, Department of Public Health and Community Medicine, Institute of Medicine, The Sahlgrenska Academy University of Gothenburg Gothenburg Sweden

**Keywords:** nurse, qualitative research, rheumatology

## Abstract

**Background:**

Telephone helplines are useful for improving patients' access to healthcare services and reducing the need for frequent face‐to‐face contact with healthcare professionals. Little is known about how people who phone a helpline perceive the encounter.

**Objectives:**

The aims of the present study were to describe the variation in how callers perceive their encounter with a rheumatology telephone helpline.

**Methods:**

The study had a descriptive, qualitative design and used a phenomenographic approach, comprising 27 semi‐structured telephone interviews with callers to Rheuma Direct, a rheumatology telephone helpline with specially trained nurses. The callers comprised 22 women and five men, aged 22–89 years (mean 54 years).

**Results:**

The callers phoned Rheuma Direct when they had problems obtaining answers to questions on the internet or from healthcare professionals. Three descriptive categories emerged: constructive dialogue, specialized competence and applicability. The callers perceived that it was a constructive dialogue when they were able to discuss their concerns with someone, received emotional support, felt reassured and were satisfied with the information provided. They perceived specialized competence when the nurses were experienced and skilful, the advice provided complemented previously received information and when they had more knowledge after the call. The callers perceived that Rheuma Direct had applicability because it was easy to access and they could make different choices before, during and after the telephone call.

**Conclusions:**

Callers to a rheumatology telephone helpline perceived it as a valuable complement to other sources of information, and felt that it could provide them with the tools to manage their disease better, as well as future contacts with healthcare professionals.

## 1 INTRODUCTION

Rheumatic diseases or musculoskeletal conditions are usually progressive and associated with chronic pain. They are also leading causes of morbidity and disability, giving rise to enormous healthcare costs (World Health Organization, 2018). Telephone helplines have been recognized as a useful means of improving access to healthcare services and reducing the need for frequent face‐to‐face contacts with healthcare professionals (Royal College of Nursing, 2012). A rheumatology telephone helpline can provide advice and support for people with rheumatic diseases but cannot always replace clinical appointments (Scrivo et al., 2014). One of the challenges of telephone consultations is obtaining a precise description of the caller's problem. Therefore, telephone helpline nurses develop skills that enable them to manage interactions with callers in order to compensate for the lack of visual cues. The quality of information is important for gaining callers' trust (Pettinari & Jessop, 2001). Callers experience safety and satisfaction when they perceive that they are the most important person in the conversation and communication with the telephone nurse is good (Holmström, Nokkoudenmäki, Zukancic, & Sundler, 2016).

Rheumatology telephone helplines are appreciated by patients (Hughes, 2003; Hughes, Carr, Huggett, & Thwaites, 2002) and can provide immediate information, advice and emotional support (Hughes, 2003). The reasons why people call a helpline include a need to discuss changes in their condition, their symptoms or to receive information on diagnosis, medications and blood test results (Brown et al., 2006). Information on rheumatic diseases is often difficult to understand or frightening, so additional support and education are often necessary (Drăgoi et al., 2013). Educational needs vary in accordance with individual characteristics such as gender, age, educational background and disease duration. Therefore, the information and education should be tailored to individual patients’ needs (Drăgoi et al., 2013). Providing information is central when helping patients to play a greater role in the treatment of their chronic condition. Information about the condition, treatment or how to avoid a deterioration would help patients to perform self‐care and play a greater role in their care (Department of Health, 2011).

The World Health Organization has presented evidence that empowering initiatives lead to positive health outcomes (Wallerstein, 2006). The basis of empowerment is meeting each individual's needs. An empowerment approach involves helping individuals to learn how to think critically and make informed decisions (Anderson & Funnell, 2010). Traditional information provided to individuals by healthcare professionals does not automatically constitute empowerment but, on the other hand, individuals cannot be empowered without information (Piper, 2010). Telephone helpline nurses can promote self‐care through individual advice but also give the callers an opportunity to discuss reflections and feelings (Ström, Marklund, & Hildingh, 2009). However, little is known about how people calling a helpline perceive the encounter. The objective of the present study was to describe the variation in how callers perceive their encounter with a rheumatology telephone helpline.

## 2 METHODS

The study had a descriptive, qualitative design with a phenomenographic approach. Within phenomenography, the focus is on how people perceive a certain phenomenon or aspect of the world. In the analysis, attention is directed to “how” the phenomenon is perceived and describing the variation in perceptions (Marton & Booth, 1997; Sjöström & Dahlgren, 2002).

### 2.1 Rheuma Direct

Rheuma Direct (RD) is a telephone helpline manned by specially trained nurses with a university education on rheumatic diseases and many years of experience in rheumatology care. RD is funded by the Swedish Rheumatism Association and Spenshult Research and Development Centre in Sweden. All nurses employed by RD are bound by professional secrecy. The purpose of RD is to provide comprehensive and accurate information about rheumatology, available treatments, research and developments in the field; and to motivate and empower the callers to perform self‐care. Contact with RD is available to the general public, which means that anyone can phone or send an email to RD, whether or not she/he has a diagnosis or is a relative, student and so forth. The call is free of charge. The callers are anonymous, which makes it impossible to document the call in the healthcare journal system. In 2016, RD was open on four afternoons a week and 1,142 people (79% women and 21% men) made contact by telephone or email. The average length of the telephone calls was about 14 min. There was a huge variety of questions, but the most common concerned symptoms, diagnosis, disease development and various forms of treatment.

### 2.2 Subjects

The inclusion criteria were that the subjects had to have phoned RD within the period January–April 2016. Twenty‐seven callers (22 [81.5%] women and five [18.5%] men), ranging in age from 22 years to 89 years (mean 54 years), gave their informed consent to be interviewed and were included in the study (Table 1). The callers were from 10 different geographical areas of Sweden; 22 (81.5%) of them had been born in Sweden and five (18.5%) in other European countries.

**Table 1:** Gender and age of the callers (*n* = 27)

Callers code	Sex	Age (years)
C 1	Woman	38
C 2	Woman	46
C 3	Woman	64
C 4	Woman	27
C 5	Woman	42
C 6	Man	35
C 7	Woman	62
C 8	Woman	33
C 9	Man	68
C 10	Woman	30
C 11	Man	63
C 12	Man	63
C 13	Woman	47
C 14	Woman	40
C 15	Woman	76
C 16	Woman	56
C 17	Woman	60
C 18	Woman	22
C 19	Woman	41
C 20	Woman	33
C 21	Woman	75
C 22	Woman	54
C 23	Woman	67
C 24	Woman	73
C 25	Woman	69
C 26	Man	89
C 27	Woman	86

### 2.3 Procedures

The data were collected by means of semi‐structured telephone interviews. A consecutive sample was used, which meant that during seven predetermined afternoons, all individuals who phoned RD were asked at the end of their call if they would like to participate in a study and be interviewed by a researcher within 1–2 weeks. The nurses gave the callers verbal information and sent them written information. Those who agreed to be interviewed provided the nurses with their name and telephone number. When the researcher contacted the callers, they gave them more detailed information about the background, objective and method of the study, in addition to assuring them that the data, as well as their contact information, would be treated confidentially. Some callers wanted to be interviewed immediately after receiving the verbal information, whereas with others a date and time for the interview were agreed. Informed consent was obtained verbally in connection with the telephone interview. Two pilot interviews were conducted to test the relevance of the questions in relation to the objective of the study. After a quality assessment, it was decided to include the pilot interviews in the data material.

Each interview lasted 15–30 min and was recorded using a digital voice recorder. The focus of the semi‐structured interviews was to engage in an open conversation, in order to deepen the understanding of how the callers perceived their encounter with a rheumatology telephone helpline. The following examples of opening questions formulated by the RD nurses were aimed at ensuring that similar data were obtained from all callers:

- “What did you think about the conversation?”
- “How did you perceive the content of the conversation?”
- “How did you feel after the conversation?”.

A better understanding of the views expressed by the callers was obtained by means of follow‐up questions such as: “How do you mean?” or |”What do you have in mind when you say. ..?”.

### 2.4 Data analysis

The interviews were transcribed verbatim. The data analysis was carried out in several steps, in accordance with the phenomenological method. In the first step, the main researcher and the coresearchers read the interview texts several times to become familiar with and obtain an overall impression of the data (familiarization). Each interview was processed by searching for statements corresponding to the objective of the study (condensation). The findings were then analysed to identify similarities and differences between the statements (comparison). Subsequently, the statements were grouped into different categories based on their characteristics (grouping), and the researchers tried to identify and describe the main similarity of the perceptions within each respective category (articulating). The next step was deciding on an appropriate descriptive label for each category (labelling). The last step in the analysis was comparing the categories in terms of similarities and differences, to ensure that each category had a unique character and was at the same level of abstraction (contrasting). Throughout the analysis, there was a constant back‐and‐forth movement between the different steps (Dahlgren & Fallsberg, 1991; Marton & Booth, 1997; Sjöström & Dahlgren, 2002).

### 2.5 Ethical approval

All parts of the study were performed with ethical approval from the regional ethical review board in Lund (no.: 2015/495). The callers were informed verbally and in writing about the study, and that participation was voluntary. They were assured of confidentiality and that they could withdraw at any time without having to justify their decision. Immediately after the interviews, the callers were given an opportunity to discuss any feelings or thoughts that might have arisen as a result of the dialogue.

## 3 RESULTS

Three descriptive categories, comprising eight perceptions, revealed how the callers perceived their contact with the rheumatology telephone helpline. The telephone conversation was considered to be a constructive dialogue based on specialized competence, and the helpline was perceived as having applicability. The quotations below illustrate both the uniqueness and the variation of the various perceptions.

### 3.1 Constructive dialogue

This descriptive category comprised three perceptions: emotional support, safety and satisfaction. A constructive dialogue implied *emotional support* in the form of being listened to and able to discuss one's concerns with someone who could confirm or refute them. The opportunity to discuss questions with a person who possesses expert knowledge of the area provided a sense of *safety*, and being encountered with understanding and respect gave rise to *satisfaction*.

#### 3.1.1 Emotional support

The callers perceived that being listened to and taken seriously during the personal contact with a nurse provided emotional support that gave them strength and energy. They also perceived emotional support when receiving confirmation of something they had thought, read, planned or done. The callers were provided with tools for moving forward, which increased their motivation to perform certain activities, such as different types of self‐care and being more assertive in their contacts with the healthcare services:

It's not that the person at the other end of the phone tells me that I have this and that, but rather that she/he encourages me to go on. (C11)

The conversation with the nurse was perceived as a positive dialogue, in which the nurse acted as a sounding board that could either support or reject what the caller had, for example, read on the internet or been told by friends or healthcare professionals:

It's important to have someone to test my ideas against. (C6)

I felt I needed to talk to someone. (C10)

I felt a little encouraged to try to get another appointment with my GP. (C23)

#### 3.1.2 Safety

The callers perceived that a conversation with someone who had time to listen and understood what they were talking about provided a sense of safety. It was also reassuring to know that the nurse probably had more knowledge than they had:

You go around harping on things and when you can't let go of them you tend to exaggerate them, and I definitely had a different feeling after the conversation. (C16)

The answer that callers received from the nurse calmed them, as it provided an explanation and was fairly similar to what they had found on the internet, or what the rheumatologist had told them. Such a confirmation instilled a sense of safety as well as confidence and hope. The peace and sense of safety that they perceived after the conversation enabled them to let go of certain thoughts:

You even feel a bit calmer when you hear several people saying the same thing. (C4)

I felt calm and decided how I wanted to proceed after the conversation. (C8).

I felt that she [the nurse] at the RD gave me confidence, so that I could let go of my worries. (C1)

#### 3.1.3 Satisfaction

The callers perceived satisfaction with the harmonious conversation with the nurse and being encountered in a friendly manner, but also with the fact that the nurse took an active interest in them and made efforts to be helpful by providing clear and informative answers. They perceived that the nurse was understanding, exhibited an interest in and respect for them as a person and took them seriously, which created both joy and energy:

And in some way, she [at RD] gave me energy that I didn't have before I phoned. (C 19)

The callers also perceived satisfaction and relief when they received detailed, clear and adequate guidance and reasoning about their worries:

Somebody who listened and didn't interrupt or cut you down to size about being in the wrong. (C9)

Talking to someone who took a holistic view of the human being and not just their physical symptoms was satisfying:

That there is someone who really listens and maybe thinks outside the body. (C19)

Believe me, I was so happy and satisfied after the conversation. (C15)

I think the information was sufficient and I will not bother to find out any more about it. (C3)

However, one caller considered that the conversation with the nurse was unsatisfactory because the answers did not contain more information than what she/he already knew.

### 3.2 Specialized competence

This descriptive category comprised three perceptions: experience and skill; increased knowledge; and a source of complementary information. Specialized competence meant being encountered by a nurse who exhibited *experience and skill* as well as the impression of gaining *increased knowledge*. The specialized knowledge imparted by the nurse was perceived as a valuable *source of complementary information* to that obtained from, for example, healthcare professionals or the internet.

#### 3.2.1 Experience and skill

The callers perceived that the nurse exhibited experience and skill, which made them feel confident. They realized that highly specialized knowledge is required to answer the many different questions posed by callers to RD. The perception that the nurses possessed expert knowledge not available at, for example, healthcare centres made it natural for the callers to turn to RD when they had specific questions about rheumatic diseases:

They know what they are talking about [at RD]. Knowledge means a lot. (C4)

I felt I was talking to a knowledgeable person. (C25)

They have top‐level expertise. (C14)

However, some callers perceived that the nurse did not provide a diagnosis and was unable to explain the reason for certain symptoms or where to find the most skilled rheumatologist:

The advice I received was very basic, that I should go to the healthcare centre, can write my own referral and so on. I've been doing that for several years, I already knew these things. (C5)

#### 3.2.2 Increased knowledge

The callers perceived gaining increased knowledge and clarity during the conversation with a nurse when provided with easily understandable information and guidance about different options for dealing with their situation. The answer could be to wait for a while before seeking further help from the healthcare service and to try to find a balance between rest and physical activity, but they could also be encouraged to insist that someone in the healthcare service should be listening to them:

RD is a good form of first aid. (C6)

The callers wanted to improve their knowledge and arguments, in order to speed up healthcare processes. Some callers contacted RD to obtain more knowledge shortly before an appointment with their doctor or after the visit, to obtain help to understand what their doctor had said. The conversation with the nurse provided them with tools that gave them greater confidence in their knowledge, thus enabling them to state and discuss their views with their doctor. They also found it easier to be firm, as the doctors could not ignore their factual knowledge. The callers perceived that they were listened to by healthcare professionals when they had greater knowledge and could argue their case. Some callers who considered that they did not obtain more knowledge or a solution to their problem during the conversation with the RD nurse nevertheless perceived receiving confirmation that what they had done was correct. Furthermore, they reported enhanced motivation to continue with self‐care and search for answers:

It strengthened me in my continuing process to know a little more about how to proceed. (C11)

I probably understood more because it was explained in greater detail and in words that were easier to understand. (C15)

After the conversation, I felt comfortable saying [to the doctor] that I did not want that medication. (C10)

#### 3.2.3 Source of complementary information

The callers perceived that what emerged during the conversation with the nurse complemented and was in line with what doctors had told them or what they had read on the internet, but with the difference that the language used was simpler, thus providing a clearer explanation of various issues. They considered it important to have information repeated to them by an independent person not involved in their care. This made it easier to assimilate the information, as well as to trust and understand what the doctors had told them, thus helping them to manage their condition:

It's safer to consult your own doctor but sometimes you need a second opinion. That is when RD is important. (C6)

The conversation with the nurse was perceived as an important source of complementary information, where all sorts of questions could be asked and the whole human being was in focus:

I think the rheumatologist focuses a great deal on the physical aspects and therefore it feels good to receive complementary information. (C4)

If I read something on the internet, maybe I want to know more about it. About something specific, and then it's good to be able to ask additional questions when talking on the phone. (C17)

Have not found anyone else in the healthcare service who I can ask such questions as to RD. (C1)

### 3.3 Applicability

This descriptive category comprised two perceptions: accessibility and possibility to choose. Applicability meant perceiving easy *accessibility* to RD whenever the need arose. There was the *possibility to choose* how and when to make contact, which questions to ask, which information to share and which actions to take after the conversation.

#### 3.3.1 Accessibility

The callers perceived that RD was accessible due to generous operating hours and the simplicity of making contact by telephone or email. They described the contact as more straightforward than that with the healthcare service or public authorities, as RD had no “nurses who acted as gate keepers” (C25). They appreciated receiving immediate answers via email and telephone, as well as not having to wait a long time in a telephone queue. However, one caller considered that the answer from the RD nurse took too long (4 days) and had expected a quicker response. The fact that it was accessible to all members of the public, irrespective of whether or not the caller had a rheumatism diagnosis or pain themselves, or wanted information about the rheumatic disease or pain suffered by a close relative or friend, was perceived as valuable:

It's very good for relatives and friends, because they have no doctor or nurse who they can phone if they are concerned about someone's pain or rheumatic disease. (C6)

When a question arises, you think about it for a long time and try to solve the problem, but in the end you just want a response or advice. And you want it immediately because you have been frustrated for such a long time that you want to talk to someone now, not in two weeks' time. (C 22)

When you phone the rheumatologist, they book a time on a day when they will return your call, but with RD you can phone directly and ask. (C3)

#### 3.3.2 Possibility to choose

The callers perceived that they had the possibility to choose to contact RD by telephone or email on a day and at a time that suited them best:

When you have a question and want an immediate answer or help and there's nowhere else to phone, RD is super because I can put my question to them and get it answered. Thus, I get a quicker response than if I tried to phone, for example, a doctor. (C22)

The callers also perceived that they had the possibility to choose whether to remain anonymous, as well as how many personal details, such as their name, age and diseases, to reveal. They appreciated being able to put questions that were important to them to the RD nurse. Depending on the outcome of the dialogue, the callers either pursued the action discussed during the conversation, continued to search for an answer or stopped pondering about it and went on with their life:

I ask about what I find interesting at the time and hope the RD can give me an answer. (C7)

This time, I emailed instead because I thought it was easier to send exactly what [the information] I wanted. (C4)

My friend received the same advice from her/his doctor and now when the person at RD said the same thing as the doctor, the friend acted on the advice. (C6)

## 4 DISCUSSION

The study explored variations in how callers perceived a rheumatology telephone helpline. They used the helpline when they had problems in obtaining answers to their questions through the internet or from healthcare professionals. They perceived their encounter with the rheumatology helpline nurses as a constructive dialogue based on specialized competence, and that the helpline had applicability due to easy access. This result is in line with the results of previous studies on various telephone helplines (Hughes, 2003; Hughes et al., 2002; Ström et al., 2009). The callers perceived that they were listened to and taken seriously during the dialogue and were satisfied when the nurse was understanding, exhibited an interest in and respect for them as a person, and treated them seriously, which is also in line with the findings of a previous study (Ström et al., 2009). This highlights the importance of interaction in the dialogue and callers' participation in decision making.

It has been shown that rheumatology nursing has an impact on all domains of patients' health, and that the most commonly measured outcomes relate to disease activity, clinical effects, pain, functionality and satisfaction in life (Minnock et al., 2018). This was also true of the present study, as the callers considered it important that the dialogue was characterized by a holistic view of them as individuals and not just by the physical problems they presented. The emotional support gave the callers strength and energy. The nurse served as a sounding board that either supported or rejected what the caller had read or been told. Previous studies have revealed that empowerment interventions such as patient education lead to improved knowledge of the disease for patients with chronic conditions and increase their level of self‐care (Chen & Li, 2009). However, although the rheumatology telephone helpline is not intended to provide patient education or counselling, it was perceived as an important source of information by many of the callers. A conversation with someone who has knowledge, time to listen and understands what the callers are talking about provides a sense of safety and satisfaction. This is in line with the findings of a previous study demonstrating that when the communication is perceived as good and the caller's perspective is the focus, the caller expresses feelings of safety and satisfaction (Holmström et al., 2016). The nurse's response can reassure callers by providing them with an explanation for their concerns. When a person perceives information as reliable, it increases the possibility that it will help her/him to perform self‐care and play a greater role in managing her/his chronic condition (Department of Health, 2011). However, self‐care demands constant planning and managing of daily life to obtain control—that is, a combination of processes carried out over a period of time (Arvidsson, Bergman, Arvidsson, Fridlund, & Bengtsson Tops, 2011). It is therefore necessary that rheumatology telephone helpline nurses find ways of checking a person's level of understanding, as only then can they support and empower a person to engage in self‐care.

In the present study the callers felt that they obtained increased knowledge and clarity during the dialogue, when the nurse gave them easily understandable information and guidance adapted to their knowledge level. The dialogue provided callers with tools that gave them greater confidence in their own knowledge. The empowerment approach (Anderson & Funnell, 2010) employed by the RD nurses helped some of the callers to think critically and make informed decisions. An earlier study also showed that callers wanted to be allowed to participate in the dialogue and decision making, and did not consider the helpline just as a source of information (Ström et al., 2009). A nurse‐led telephone intervention combining emotional support, health information and decision‐making assistance has the potential to reduce health problems and increase the satisfaction of patients and their families when managing rheumatic diseases (Ramelet et al., 2017). Communication skills are essential when providing telephone nursing to callers, but a person‐centred approach is equally important for optimal care.

The majority of callers perceived that the nurse provided complementary information to that which they had previously obtained, as well as a clearer explanation of various issues. It was important for the callers to have the information repeated by a person with experience and competence who was not involved in their care. The information often helped the caller to decide what to do next. However, decision aids differ from information obtained through standard health education because they provide a detailed and personalized focus on options and outcomes for the purpose of preparing the individual for decision making (Stacey et al., 2014). If callers obtain disease and therapy management support or can discuss side effects and/or symptom aggravation, a large proportion of them will not need to search for further health services (Ferreira, Marques, Mendes, & da Silva, 2015). A rheumatology telephone helpline, involving nurses with specialized knowledge about rheumatic diseases, the impact such diseases have on peoples' lives and how to engage in shared decision making and goal setting, can provide patients with education, information and tools to perform appropriate self‐care. This, in turn, empowers patients to become more involved in their care, thereby improving patient safety.

The callers perceived it as easy to access the helpline whenever the need arose. They appreciated the possibility of choosing actions depending on the outcome of the dialogue, performing the self‐care discussed during the conversation with the nurse, continuing to search for an answer, or ceasing to ponder and getting on with their lives. A previous study demonstrated that callers would have consulted their general practitioner if the telephone helpline had not been available. A rheumatology telephone helpline can thus save resources and reduce costs (Hughes et al., 2002). The challenge for clinical units providing rheumatology telephone helplines is to ensure that this valuable service is recognized and secure funding for it. Further studies are needed to find out how the telephone conversation affects callers in the long term, and if a rheumatology telephone helpline is a cost‐saving resource for both the patient and the healthcare system.

### 4.1 Strengths and limitations of the study

The intention was to carry out at least 25 interviews, to capture a variety of perceptions. On seven previously agreed afternoons, 27 callers were recruited for the study. The sample size had not been decided beforehand but was determined by the callers' ability to provide rich and in‐depth data. The interviews presented a comprehensive description of how callers perceived their encounter with a rheumatology telephone helpline. Twelve callers declined to participate in the study for various personal reasons. Twenty‐seven callers were interviewed by the same researcher, which strengthened credibility. The interviews took place 1–3 weeks after the phone call to RD. The questions in the semi‐structured interview guide were developed in collaboration with RD nurses and based on their experience of such telephone calls. One of the nurses was also a research partner, in addition to being a patient with 30 years' experience of rheumatoid arthritis. A limitation of the study was that the interview guide was not raised in cooperation with previous callers. However, the two pilot interviews demonstrated their relevance. The telephone interviews were characterized by openness and flexibility towards the callers. The fact that the interviews were conducted by telephone was also a limitation but this made it possible to interview all callers who agreed to participate, irrespective of where they lived. The analysis process was carried out in collaboration with all the researchers but without any external reviewers, which was a limitation in terms of deciding on perceptions and the categories. However, dependability was strengthened by the fact that the researchers first analysed the data separately and then compared their findings. To strengthen confirmability, the researchers considered and critically reflected on their preunderstanding. The detailed presentation of the results, together with appropriate quotations, enhanced transferability. The researchers therefore consider that the results will be of interest to healthcare professionals and also to people suffering from rheumatic diseases.

## 5 CONCLUSION

In the present study, the callers perceived their encounter with a rheumatology telephone helpline as a constructive dialogue based on specialized competence, and that the telephone helpline had applicability. The constructive dialogue provided emotional support and a sense of safety. Being encountered with understanding and respect gave rise to satisfaction. The callers perceived that the nurse exhibited experience and skills, which enabled them to gain increased knowledge, described as valuable complementary information to that obtained from healthcare professionals or on the internet. The callers reported that it was easy to access the rheumatology telephone helpline whenever the need arose and that they were free to make their own decisions before, during and after contact. Further studies are needed to determine how phoning the rheumatology telephone helpline affects the caller in the long term and if such a helpline is a cost‐saving resource for both the individual and the healthcare system.

## FUNDING INFORMATION

Funding was obtained from the Swedish Rheumatism Association (grant number: R‐558491) and from the Spenshult Research and Development Centre.

## CONFLICTS OF INTEREST

The authors declare that there are no conflicts of interest that could be perceived as prejudicing the impartiality of the research reported.
